# Germline Genetic Associations for Hepatobiliary Cancers

**DOI:** 10.1016/j.jcmgh.2023.12.010

**Published:** 2023-12-30

**Authors:** Perapa Chotiprasidhi, Angela Karina Sato-Espinoza, Kirk J. Wangensteen

**Affiliations:** Division of Gastroenterology and Hepatology, Department of Medicine, Mayo Clinic, Rochester, Minnesota

**Keywords:** *BRCA2*, Genetic Testing, Personalized Medicine, Hepatocellular Carcinoma, Cholangiocarcinoma

## Abstract

Hepatobiliary cancers (HBCs) include hepatocellular carcinoma, cholangiocarcinoma, and gallbladder carcinoma, which originate from the liver, bile ducts, and gallbladder, respectively. They are responsible for a substantial burden of cancer-related deaths worldwide. Despite knowledge of risk factors and advancements in therapeutics and surgical interventions, the prognosis for most patients with HBC remains bleak. There is evidence from familial aggregation and case-control studies to suggest a familial risk component in HBC susceptibility. Recent progress in genomics research has led to the identification of germline variants including single nucleotide polymorphisms (SNPs) and pathogenic or likely pathogenic (P/LP) variants in cancer-associated genes associated with HBC risk. These findings emerged from genome-wide association studies and next-generation sequencing techniques such as whole-exome sequencing. Patients with other cancer types, including breast, colon, ovarian, prostate, and pancreatic cancer, are recommended by guidelines to undergo germline genetic testing, but similar recommendations are lagging in HBC. This prompts the question of whether multi-gene panel testing should be integrated into clinical guidelines for HBC management. Here, we review the hereditary genetics of HBC, explore studies investigating SNPs and P/LP variants in HBC patients, discuss the clinical implications and potential for personalized treatments and impact on patient’s family members, and conclude that additional studies are needed to examine how genetic testing can be applied clinically.

**Summary**

This is a review of the single nucleotide polymorphisms and pathogenic and likely pathogenic germline variants that have been linked to hepatocellular carcinoma, cholangiocarcinoma, and gallbladder cancer, the implications on management of patients, and the gaps in knowledge that should be addressed in future studies.

## Background

Hepatocellular carcinoma (HCC), cholangiocarcinoma (CCA), and gallbladder cancer (GBC), collectively known as hepatobiliary cancers (HBCs), constitute a significant portion of the global burden of liver and biliary tract cancers. Primary liver cancer is the sixth most common cancer and is the third leading cause of cancer-related deaths. HCC is the most prevalent primary liver cancer, accounting for 75%–85% of all cases.^1^

HCC usually arises in the setting of cirrhosis, the end-stage of necroinflammatory injury of the liver. Therefore, the main risk factors for HCC are conditions that lead to cirrhosis, including chronic hepatitis B and hepatitis C virus (HBV/HCV) infection, metabolic syndrome, excessive alcohol intake, exposure to environmental toxins (eg, aflatoxin), and inherited genetic syndromes such as Wilson disease, glycogen storage disorder, and hemochromatosis.^2^, ^3^, ^4^, ^5^, ^6^, ^7^, ^8^

CCA, a malignancy arising in the bile ducts, represents about 10%–15% of primary liver cancers.^1^^,^^9^ The main risk factors are chronic inflammation of the bile ducts, often caused by conditions such as primary sclerosing cholangitis, fibropolycystic liver disease, and cholelithiasis. Additional risk factors include alcohol and tobacco use, infection with liver flukes, HBV, HCV, human immunodeficiency virus, *Helicobacter pylori*, and metabolic syndrome.^10^, ^11^, ^12^, ^13^, ^14^, ^15^, ^16^ CCA is often detected in advanced stages, and outcomes are generally poor.^17^

GBC is rare, accounting for less than 1% of all cancer-related deaths.^1^ Carcinogenesis of the gallbladder is primarily due to prolonged irritation and inflammation of epithelial cells from gallstones, primary sclerosing cholangitis, chronic infections with salmonella, *H pylori*, congenital biliary cysts, obesity, and carcinogen exposure.^18^, ^19^, ^20^, ^21^, ^22^, ^23^, ^24^, ^25^, ^26^, ^27^ GBC incidence varies globally, with a higher prevalence in some regions of South America and Asia.^28^ GBC is often lumped together with CCA in studies and termed biliary tract cancer (BTC).

Current guidelines recommend surveillance in people who are at elevated risk of cancers of the liver. To prevent HCC or to catch it at early stages, screening tests are recommended for all patients with cirrhosis and are tailored on the basis of chronic infection with hepatitis B or C infection, advanced age, and geographic origin from high-risk areas.^29^ The main tools used in HCC surveillance are imaging with ultrasound and alpha-fetoprotein measurement.^29^^,^^30^

Guidelines for CCA and GBC recommend screening for people with primary sclerosing cholangitis, gallbladder polyps, and chronic biliary inflammation.^31^ Ultrasound, computed tomography, and magnetic resonance imaging scans are all used for screening.^31^ For patients with suspected distal extrahepatic CCA (EHC), endoscopic retrograde cholangiopancreatography with either fine-needle aspiration or brush cytology is used for screening and diagnosis.^32^

A family history of HCC independently increases HCC risk by more than 2.5 times, as seen in populations in Italy^4^ and Texas.^5^ In addition, a family linkage study shows that genetics play a role in up to 40% of HCC cases in individuals with chronic HBV infection.^33^ A study in Taiwan revealed a synergy between HBV infection and family history, with family history accounting for a risk attribution fraction of 0.59.^34^ Familial clustering of GBC has been described several times.^35^^,^^36^ The Swedish Cancer Registry has demonstrated familial risk for HCC and GBC, with standardized incidence elevated at 2.60 and 2.76, respectively.^37^ Regarding CCA, limited research has been conducted on family clustering and case-control studies. Overall, the findings underscore familial factors in HBC risk, warranting investigations into genetic associations.

Germline genetic testing has become more accessible and affordable in the clinic, with significant implications for patients and their families. Blood or saliva DNA is tested for pathogenic or likely pathogenic (P/LP) germline variants associated with cancer risk according to American College of Medical Genetics criteria, which are generally loss-of-function mutations.^38^ Genetic testing for cancer-associated genes is recommended by clinical practice guidelines for breast, colon, ovarian, prostate, and pancreatic cancer,^38^, ^39^, ^40^ but not yet for HBC. Guideline-directed testing misses up to 50% of P/LP germline genetic variants in patients with solid cancers, which has led some to advocate for universal testing in cancer.^41^ As we will discuss, germline variants are detectable in patients with HBC. This review examines evidence of single nucleotide polymorphisms (SNPs) and P/LP germline variants detected in HBC, their possible role in pathogenesis, and the clinical implications for therapy and risk management in family members.

## Introduction to Germline Genetic Association Studies

Genome-wide association studies (GWAS) and next-generation sequencing (NGS) technologies have been pivotal in identifying both common and rare genetic variants associated with cancer risk, respectively. Large-scale population cohorts, such as the UK Biobank, All of Us, BioBank Japan, and the Penn Medicine BioBank, serve as invaluable repositories of health and genetic data, which are useful for identifying patterns of heritability across diverse demographic groups.^42^ The following sections will break down the details regarding the differences between GWAS and rare variant analysis in relation to HBC.

In general, SNPs are variants present in populations at rates >1% and are detected in GWAS that compare millions of SNPs in hundreds or thousands of cases versus matched controls.^43^ The SNPs can be found anywhere in the genome including within gene coding sequences or in non-coding sequences and sometimes reside thousands of bases from the nearest gene. GWAS have detected SNPs associated with HBC. Usually, the mechanisms leading to cancer risks are not determined directly by GWAS; additional studies are needed to define how the genetic change affects gene expression levels or gene function in cancer development. The risk allele SNP can be found in 1 copy in heterozygous or 2 copies in homozygous persons, usually associated with incrementally increasing risk. Polygenic risk scores (PRS) are a composite measure combining the individual risk alleles to estimate an individual’s genetic predisposition to a particular trait or disease.^44^ PRS offer personalized risk assessments, potentially enabling early intervention and tailored treatment strategies for cancer screening or prevention based on their level of genetic risk.

Rare variant analysis focuses on variants that are found at rates lower than 1% and often at rates of much less than 0.1% in the general population.^45^ Rare variants are detected using NGS by sequencing gene exons.^46^ Rare variant studies in HBC have generally focused on genes known to be linked to cancer predisposition syndromes such as *BRCA1*, *BRCA2*, and the Lynch-syndrome genes (*MLH1*, *MSH2*, *MSH6*, *PMS2*) among others, which are associated with hereditary breast and ovarian cancer syndrome and predisposition to colon and uterine cancer, respectively. These variants usually disrupt gene function by causing a premature truncation or causing a mutation in a crucial nucleotide. The mechanism of the gene variant in causing disease is often a direct consequence of a loss-of-function of the gene. Rare variants are usually found to be heterozygous and are inherited in an autosomal dominant pattern. Tumors often undergo loss-of-heterozygosity of these genes, meaning that the allele unaffected by the germline variant is lost or mutated in the tumor.^47^ Many of the known cancer-associated genes are involved in DNA repair pathways. One promising therapeutic approach involves the use of poly(adenosine diphosphate ribose) polymerase (PARP) inhibitors in patients with germline defects in homologous recombination-DNA damage repair (HR-DDR) genes. These HR-DDR genes, including *BRCA1*, *BRCA2*, *ATM, CHEK2*, and Fanconi’s anemia genes (eg, *FANCA*, *BRIP1*), are associated with increased susceptibility to PARP inhibitors in various cancer types, such as breast, ovarian, prostate, and pancreatic cancers.^48^, ^49^, ^50^, ^51^, ^52^ PARP inhibitors have many potential mechanisms, but one way that the PARP inhibitor works is by tethering PARP complexes to unrepaired DNA ends, leading to cell death.^53^ These inhibitors may be effective even if only one allele of an HR-DDR gene is mutated in tumors.^53^

Both GWAS and rare variant studies depend on adequate sample size of the population affected with cancer and the population of matched controls.^54^ Also, results may uncover different associations depending on whether the control population is matched for environmental risk factors for HBC, especially for cirrhosis in the case of HCC. For example, unless the control population is matched for cirrhosis severity with the HCC group, variants associated with HCC may simply worsen liver disease as a precursor to cancer rather than directly contributing to carcinogenesis. Finally, different disease etiologies may be linked to different genetic associations, suggesting specific interactions between environmental factors and genetic risk.

## Genetic Associations for Hepatocellular Carcinoma

### Single Nucleotide Polymorphisms Associated With Hepatocellular Carcinoma

Because prolonged liver injury and cirrhosis precede most HCCs, the genetic risks associated with steatosis, elevated liver enzymes, and cirrhosis will also generally increase the risk of HCC. Several large GWAS in liver disease included many thousands of cases of steatosis,^55^, ^56^, ^57^ elevated alanine aminotransferase,^57^, ^58^, ^59^ and cirrhosis^56^^,^^58^^,^^59^ versus healthy controls and have identified dozens of SNPs associated with liver diseases. These studies have found that people with a high index of genetic risks, who score high for polygenic risk of liver disease, also have elevated risks of HCC.

Here, we focus on GWAS studies that have investigated genetic risks in patients with HCC versus controls with liver disease who do not have HCC. Table 1 lists the gene nearest to the SNP, the accession number, effect size, *P* values, and other details of these studies. These studies are designed to identify genetic risk of progression to HCC among people with preexisting liver disease. Many, but not all, of the associations for HCC overlap within the SNPs that have emerged in liver diseases GWAS. We discuss the SNPs according to the specific environmental factors studied in GWAS.

**Table 1:** List of Genome-wide Association Studies of HCC

Gene	SNP	Risk allele	OR (95% CI)	*P* value	Type of cancer cases	Type of controls	No. of subjects				Ethnic group/country	References
							Discovery phase		Replication phase			
							Cases	Controls	Cases	Controls		
*CDK14*	rs10272859	G	1.28 (1.18–1.38)	9.46 × 10^−10^	HBV+ HCC	HBV	537	737	3796	2544	Chinese	Li et al^60^
*DEPDC5*	rs1012068	G	1.75 (1.51–2.03)	1.27 × 10^−13^	HCV+ HCC	HCV	212	765	710	1625	Japanese	Miki et al^61^
*GRIK1*	rs455804	C	0.84 (0.80–0.89)	5.24 × 10^−10^	HBV+ HCC	HBV	1538	1465	4431	4725	Han Chinese	Li et al^62^
*HLA* class I region	rs2523961	G	1.91 (1.56–2.37)	6.42 × 10^−10^	HBV+ HCC	HBV	473	516	153	614	Japanese	Sawai et al^63^
	rs1110446	C	1.93 (1.57–2.37)	2.52 × 10^−10^								
*HLA-DQ*	rs9275319	A	1.49 (1.36–1.63)	2.72 × 10^−17^	HBV+ HCC	HBV	1161	1353	4319	4966	Chinese	Jiang et al^64^
*HLA-DQA/DQB*	rs9275572	A	1.30 (1.19–1.42)	5.97 × 10^−9^	HCV+ HCC	Healthy	721	2890	673	2596	Japanese	Kumar et al^65^
*HLA-DQA1/DRB1*	rs9272105	G	1.28 (1.22–1.35)	5.24 × 10^−22^	HBV+ HCC	HBV	1538	1465	4431	4725	Han Chinese	Li et al^62^
*HLA-DQB1*	rs9274684	NR	1.54 (1.36–1.74)	7.79 × 10^−12^	HCV+ HCC	HCV	502	749	669	429	Taiwanese	Lee et al^66^
	rs9275521	NR	1.56 (1.35–1.80)	1.38 × 10^−9^								
	rs2647046	NR	1.56 (1.35–1.80)	1.42 × 10^−9^								
	rs6928482	NR	1.95 (1.73–2.19)	1.67 × 10^−29^								
	rs2856723	NR	2.68 (2.32–3.09)	2.58 × 10^−43^								
	rs9275086	NR	2.10 (1.84–2.40)	7.38 × 10^−28^								
	rs2858324	NR	2.42 (2.11–2.78)	1.09 × 10^−36^								
*IFNL2, IFNL3*	rs8107030	G	1.44 (1.28–1.62)	7.96 × 10^−10^	HCC	Non-HPB cancers	1866	195,745	NR	NR	Japanese	Ishigaki et al^67^
*KIF1B*	rs17401966	G	0.61 (0.55–0.67)	1.7 × 10^−18^	HBV+ HCC	HBV	348	359	1962	1430	Chinese	Zhang et al^68^
*MICA*	rs2596542	G	1.39 (1.27–1.52)	4.21 × 10^−13^	HCV+ HCC	Healthy	721	2890	673	2596	Japanese	Kumar et al^65^
*PNPLA3*	rs2294915	T	1.75 (1.55–1.97)	2.44 × 10^−19^	ArC + HCC	ArC	1066	844	148	1022	Austria Germany Italy Switzerland UK	Buch et al^69^
	rs738409	G	1.74 (1.54–1.97)	4.31 × 10^−19^								
	rs738409	G	1.34 (1.22–1.47)	7.30 × 10^−10^	ArC + HCC	ArC	775	1332	874	1059	Belgium France	Trepo et al^70^
	rs2281135	A	1.64 (1.39–1.95)	7.43 × 10^−9^	HCC + NAFLD + HCV	GPC	721	1997	b	b	Japan Singapore Taiwan United States	Wang et al^71^
			1.32 (1.07–1.57)	0.026^a^		CLD+ HCV						
	rs4823173	A	1.61 (1.36–1.90)	6.28 × 10^−8^^a^		GPC						
			1.30 (1.05–1.55)	0.044^a^		CLD+ HCV						
	rs2896019	C	1.60 (1.35–1.89)	3.63 × 10^−8^		GPC						
			1.27 (1.00–1.62)	0.054^a^		CLD + HCV						
		G	3.37 (2.21–5.14)	1.8 × 10^−8^	NASH-HCC	GPC	58	7672	NR	NR	Japanese	Kawaguchi^72^
*STAT4*	rs7574865	G	1.21 (1.14–1.28)	2.48 × 10^−10^	HBV+ HCC	HBV	1161	1353	4319	4966	Chinese	Jiang et al^64^
*TERT*	rs2242652	A	0.61 (0.52–0.72)	6.40 × 10^−9^	ArC + HCC	ArC	1066	844	148	1022	Austria Germany Italy Switzerland UK	Buch et al^69^
*TLL1*	rs17047200	T	2.37 (1.74–3.23)	2.66 × 10^−8^	HCV+ HCC	HCV + EOT	123	333	130	210	Japanese	Matsuura^73^
*TM6SF2*	rs58489806	T	1.88 (1.57–2.25)	3.04 × 10^−12^	ArC + HCC	ArC	1066	844	148	1022	Austria Germany Italy Switzerland UK	Buch et al^69^
	rs58542926	T	1.98 (1.64–2.40)	1.00 × 10^−12^								
	rs58542926	T	1.77 (1.52–2.07)	3.84 × 10^−1^³	ArC + HCC	ArC	775	1332	874	1059	Belgium France	Trepo^70^
*WNT3A-WNT9A*	rs708113	T	0.73 (0.66–0.81)	3.93 × 10^−10^	ArC + HCC	ArC	775	1332	874	1059	Belgium France	Trepo^70^

ArC, alcohol-related cirrhosis; CLD, chronic liver disease; NAFLD, nonalcoholic fatty liver disease; EOT, end of treatment; GPC, general-population controls; HBV, hepatitis B virus; HCV, hepatitis C virus; NASH-HCC, nonalcoholic steatohepatitis–derived hepatocellular carcinoma; NR, not reported.

a Studies that have a *P* value greater than 5 × 10^−8^.

b Studies that contain multiple prior GWAS studies that were used for their replication phase.

### Alcohol

A case-control GWAS study conducted in European populations included cases of HCC and matched controls, all with chronic alcohol consumption.^70^ There were associations for SNPs at *PNPLA3* (rs738409; odds ratio [OR], 1.34; 95% confidence interval [CI], 1.22–1.47), *TM6SF* (rs58542926; OR, 1.77; 95% CI, 1.52–2.07), and *WNT3A-WNT9A* (rs708113; OR, 0.73; 95% CI, 0.66–0.81) loci. The SNPs in *TM6SF*, rs58489806 (OR, 1.88; 95% CI, 1.57–2.25) and rs58542926 (OR, 1.98; 95% CI, 1.64–2.40), and *PNPLA3*, rs2294915 (OR, 1.75; 95% CI, 1.55–1.97) and rs738409 (OR, 1.74; 95% CI, 1.54–1.97) had previously been associated with liver disease severity in obese individuals and in people who consume alcohol and has been confirmed in a separate European study.^69^ The SNP at the *WT3A-WNT9A* locus appeared to protect from HCC in alcohol users but had no effect in other etiologies of chronic liver disease.^70^ This suggests a specific interaction for this SNP with alcohol in driving carcinogenesis.

The gene *PNPLA3* encodes adiponutrin, an enzyme with triglyceride lipase activity that plays a role in lipid metabolism. The risk allele encodes a change from isoleucine to methionine at adiponutrin amino acid 148 (I148M), which reduces hydrolase activity and disrupts ubiquitination and proteasome degradation, resulting in lipid accumulation.^74^ This accumulation can progress to hepatic steatosis, steatohepatitis, cirrhosis, and subsequently HCC. Mice with the I48M allele had normal hepatic fat levels on a chow diet but 2- to 4-fold increased liver fat level with sucrose feeding.^74^, ^75^, ^76^, ^77^ This finding correlates with what is observed in humans, where PNPLA3-I48M carriers develop steatosis in obesity but not in a lean state.^78^

The *TM6SF2* gene encodes transmembrane 6 superfamily 2 and is associated with hepatic lipoprotein export and the lipidation of very-low-density lipoprotein. The SNP associated with HCC is thought to be a loss-of-function mutation that disrupts very-low-density lipoprotein secretion.^79^^,^^80^ However, the precise mechanism leading to HCC development is unclear. In mouse models of steatosis, knockout of *TM6SF2* increases fibrosis and accelerates development and progression of HCC.^81^

*WNT3A* and *WNT9A* are members of the Wnt gene family, which is known to be pivotal in cellular proliferation, differentiation, maintenance of liver homeostasis, and facilitation of adult tissue repair.^82^^,^^83^ Further investigation is needed to define the mechanism of the SNP in protecting from HCC in people who use alcohol.

Another SNP implicated in alcohol-related HCC is at the *TERT* locus. A GWAS study involving European patients found that SNP rs2242652 (OR, 0.61; 95% CI, 0.52–0.72) was associated with a decreased risk of HCC.^69^ This finding was replicated in a Chinese study for the same SNP, reinforcing that specific SNPs in *TERT* may play a protective role against HCC development.^84^ *TERT* plays a crucial role in enzymatic telomerase synthesis, preserving genomic stability by maintaining telomere integrity. This regulatory function extends to the modulation of cellular proliferation, aging processes, and senescence.^85^^,^^86^ Longer telomeres in hepatocytes may counteract known risk factors associated with HCC such as cirrhosis, male sex, and advanced age.^69^ In HCC, the *TERT* promoter frequently undergoes somatic mutation, consequently leading to an up-regulation in *TERT* expression in tumor tissues. Overexpression of *TERT* causes cancer progression via facilitating cellular proliferation, bypassing cellular senescence, and lengthening of telomeres. Despite the germline SNP and the mutations influencing *TERT* similarly, increasing *TERT* expression and lengthening telomeres, their impact on HCC risk differs; the germline SNP contributes to reduced HCC risk, whereas somatic *TERT* mutations promote carcinogenesis. This discrepancy needs future studies to understand the exact mechanism.

### Metabolic Dysfunction–Associated Steatotic Liver Disease

A study conducted in Japan focused on individuals with metabolic dysfunction–associated steatotic liver disease (MASLD), previously called nonalcoholic fatty liver disease, and HCC arising from metabolic dysfunction–associated steatohepatitis, previously called nonalcoholic steatohepatitis, performed a GWAS including 58 metabolic dysfunction–associated steatohepatitis–HCC cases and 7672 healthy controls.^72^ The finding revealed an association between the *PNPLA3* SNP rs2896019 (OR, 3.37; 95% CI, 2.21–5.14) and the development of HCC in patients with metabolic dysfunction–associated steatohepatitis. A recent multicenter study conducted in non-Hispanic white patients with MASLD and metabolic syndrome, including HCC cases and cancer-free controls, found an association between the *PNPLA3* SNP rs738409 (OR, 1.52; 95% CI, 1.27–1.82) and an increased risk of developing HCC.^87^

### Hepatitis C

A GWAS performed in Japan included HCC cases with HCV and chronic HCV carriers without liver cancer as controls and revealed SNP rs1012068 at *DEPDC5* was associated with increased susceptibility to HCC development in individuals with HCV (OR, 1.75; 95% CI, 1.51–2.03).^61^ However, 2 additional studies in Saudi Araba^88^ and Northern Italy^89^ could not replicate this result. The *DEPDC5* gene encodes DEP domain-containing protein 5, which plays a role in mammalian targets of rapamycin signaling. The significance of this SNP is unclear without confirmation in additional studies.

A separate GWAS performed in Japan found a SNP, rs2596542, upstream of the *MICA* gene was associated with HCC in HCV-infected people (OR, 1.39; 95% CI, 1.27–1.52).^65^ This was confirmed in a Brazilian cohort.^90^ *MICA* (MHC class I polypeptide-related sequence A) belongs to the natural killer group 2D (NKG2D) receptor family, which can trigger natural killer cell–mediated cytotoxicity in response to virus-infected cells. The mechanism by which *MICA* could contribute to HCC development may be via the up-regulation of matrix metalloproteins, diminishing the antitumor effect of immune cells and facilitating immune evasion by HCC cells.^8^^,^^91^^,^^92^

Another immune-related SNP identified in a Japanese population linked to HCC development in the setting of HCV infection is SNP rs17047200 (OR, 2.37; 95% CI, 1.74–3.23) at the *TLL1* gene locus, which was significantly associated with the development of HCC after the eradication of HCV and achievement of sustained virologic response through interferon-based therapy.^73^ However, studies in Italy^93^ and Egypt^94^ could not replicate the findings. The *TLL1* gene encodes Tolloid-like protein 1, which plays a role in regulating extracellular matrix assembly and the transforming growth factor β signaling pathway.^95^ This SNP requires further confirmation.

### Hepatitis B

A SNP, rs17401966, at the *KIF1B* gene locus was involved in the pathogenesis of HCC among chronic HBV carriers in a Chinese population (OR, 0.61; 95% CI, 0.55–0.67).^68^ However, studies in Saudi Arabia^96^ and Thailand^97^ could not replicate the results. The *KIF1B* gene may promote virus infection by increasing nuclear envelope permeability.^98^ A separate Chinese GWAS study identified SNP rs455804 at the *GRIK1* gene locus may decrease risk of HCC development in HBV carriers (OR, 0.84; 95% CI, 0.80–0.89).^62^ The *GRIK1* gene encodes an ionotropic glutamate receptor.^99^ The mechanism underlying its potential involvement in cancer development and its functional role in HCC are unknown. Additional data are needed to substantiate the role of these SNPs in HCC risk.

### Summary on Associations of SNPs and HCC

Overall, several SNPs have been associated with HCC risk. SNPs in *TM6SF2* and *PNPLA3* are lipid metabolism genes that have been associated with HCC development in patients with MASLD and chronic alcohol consumption across several studies, representing perhaps the strongest association to date. These SNPs are also associated with liver disease severity. Larger studies that carefully control for liver disease severity and duration in HCC and control groups are needed to define whether these SNPs are directly associated with carcinogenesis or are simply associated with liver disease severity as a precursor to HCC. SNPs at the *WNT* and *TERT* loci may be linked to cancer formation more directly, because they are genes that are involved in the cell cycle and senescence. In addition, SNPs related to viral hepatitis and HCC tend to occur at loci related to inflammation and possibly are linked to worse necroinflammation. Most of these SNPs have not been replicated in different populations, necessitating further research to comprehend the impact on HCC development.

## Rare Variants in Cancer-Associated Genes in Hepatocellular Carcinoma

**Figure 1:**
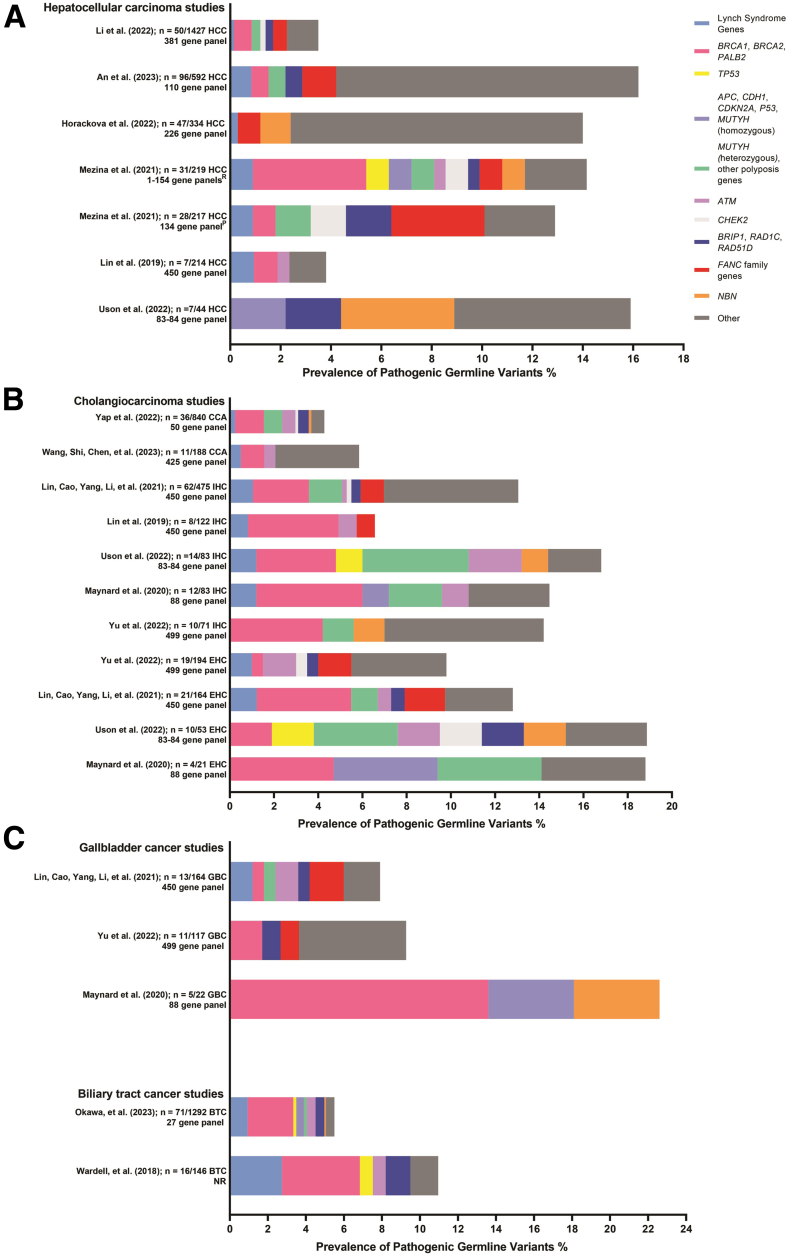
**Prevalence of pathogenic and likely pathogenic germline variants across rare variant studies in HBCs.** This chart summarizes the yield of genetic testing across studies that have included patients with (*A*) HCC (*B*) CCA, IHC, and EHC and (*C*) GBC and BTC. Biliary tract cancer studies group CCA and GBC patients into one cancer population. Lynch syndrome genes included *MLH1*, *MSH2*, *MSH6*, *PMS2*, or *EPCAM*. Other polyposis genes include *BMPR1A*, *RPS20*, *SMAD4,* or *STK11*. *FANC* family genes include *FAN1*, *FANCA*, *FANCC*, *FANCD2*, *FANCG*, *FANCI*, *FANCL*, or *FANCM*. Other groups included individuals who had *ATR*, *ATRIP*, *AXIN1*, *BAP1*, *BARD1*, *BLM*, *BUB1B*, *BUB3*, *CDC73*, *DMBT1*, *EGFR*, *ERCC2*, *ERCC5*, *ERCC6*, *ECO1*, FAM175A*, FH c.1431_1433dup*, *GALNT12*, *HOXB13*, *LIG3*, *MCPH1*, *MDC1, MLH3, MITF, MRE11A, MMP8 MSH3* (monoallelic), *NF1, NHEJ1, NTHL1, PIK3CG, PMS1, POT1, PRSS1,* and *POLQ*. ^P^ Indicates studies that use a prospective cohort of patients. ^R^ Indicates studies that use a retrospective cohort of patients.

Studies have identified P/LP variations in cancer predisposition genes in patients with HCC (Figure 1*A*). Notably, only 2 studies have used a commercialized Clinical Laboratory Improvement Amendments–certified laboratory NGS panel, resulting in the return of results for P/LP variants by a genetic counselor.^100^^,^^101^ The remaining rare variant analyses discussed in this review either used in-house sequencing or relied on preexisting databases.

A study in China used an in-house targeted NGS panel that encompasses the exons of 450 cancer-related genes. Their cohort consisted of 214 patients with HCC, 122 with intrahepatic cholangiocarcinoma (IHC), and 21 with mixed HCC-CCA.^102^ P/LP germline variants were discovered in 4.2% of the patients overall, with P/LP variants in moderate to high penetrance genes *ATM*, *BLM, BRCA1, NBN, PMS2,* and *RAD50* detected in HCC. Another study from China^103^ examined 381 cancer-associated genes in the germline of 1427 patients with HCC and discovered 3.5% of these patients carried germline variants in *ATR, BLM, BRCA1/2, CHEK2, FANCA, FANCC, FANCD2, MSH6, MLH11, MUTYH, PALB2, PMS2, RAD50,* and *SMARCA4*. The method for calling gene mutations was not described, and the specific variant allele sequences were not reported in this study.

Cohorts of prospectively and retrospectively were composed of U.S. patients with HCC who were sequenced for cancer-related genes in a commercial lab, and 11.5% and 13.7% of patients carried germline variants, respectively.^100^ The prospective cohort enrolled patients with HCC at a tertiary hospital in Philadelphia, PA, and 1.8% of patients were found to have P/LP germline variants in high penetrance genes *BRCA2*, *MSH6*, and *PMS2*. In addition, a significant enrichment of P/LP variants in moderate penetrance genes *BRIP1* and *FANCA* was observed when comparing HCC patients with general population rates. The retrospective cohort included patients referred for testing at a commercial lab in the United States, and 5.9% had germline variants in high penetrance genes *APC*, *BRCA1*, *BRCA2*, *MSH2*, and *TP53*.

A study in the Czech Republic consisted of 334 patients with HCC and 1662 controls from the general population who had their DNA sequenced with an in-house panel 226 cancer-related genes.^104^ Within the patient cohort, a total of 47 patients carried P/LP variants representing a prevalence of 14.1%. However, a noteworthy finding was that only 7 of the 334 patients (2.1%) harbored a P/LP variant in established cancer-associated genes, specifically *PMS2*, *NBN*, *FH*, or *RET*. There was a statistically significant higher frequency in P/LP variants in *NBN* and *RAD50*, which are involved in the MRE11-RAD50-NBS1 (MRN) complex, when comparing HCC cases with controls. The MRN complex is involved in genomic stability because it senses DNA damage and initiates DNA double-strand break repair.^105^^,^^106^

A multicenter study that included 205 patients with HBC, encompassing IHC, EHC, HCC, and ampullary carcinoma, found that 15.6% of patients carried germline variants.^101^ Specifically, among the 44 patients with HCC, 15.9% were identified to carry germline variants including one with a variant in the highly penetrant gene *CDKN2A* and others with variants in moderate penetrance genes *MTF*, *NBN*, and *RAD51D*.

In a study of 592 patients with HCC that were combined from The Cancer Genome Atlas study in the United States and a Korean cohort, whole-exome sequencing (WES) revealed that 16.2%, 96 out of 592 patients, carried germline variants in DDR genes. Although some of the genes are not considered clinically relevant, moderate-high penetrance variants included *BRCA1/2, BRIP1, FANCA, MLH1, MSH2, MSH6, MUTYH, PALB2, PMS2,* and *RAD1C*.^107^

An international multicenter study that was predominately U.S. and European non-Hispanic white patients focused on metabolic genes linked to HCC risk, and 556 patients with HCC and 643 controls underwent targeted exome sequencing of 64 genes.^87^ They identified a rare variant in *ABCC2* was associated with HCC risk. The *ABCC2* gene encodes a transmembrane transport protein complex (MRP2) involved in the transport of organic anions and drugs from the hepatocytes into bile. Patients with complete loss of function of this gene develop Dubin–Johnson syndrome.^108^ The mechanism by which variants in this gene may increase HCC risk is not clear.

Although overall they are rare, the detection of pathogenic variants in DDR pathway genes opens opportunities for targeted therapies. Examples include PARP inhibitors such as olaparib, which have been shown to be effective in treating cancers with *BRCA1* and *BRCA2* mutations among others.^109^ Preclinical studies, which use clinical HCC tumor tissues,^110^ xenograft mouse model,^110^ and human HCC cell lines,^107^ have suggested that HCC cells with defective homologous recombination are sensitive to PARP inhibitors. In the clinic, a patient with progressive HCC harboring a BRCA2 germline variant was administered olaparib but had disease progression.^100^ In a separate study, a patient with refractory HCC carrying an *FANCA* germline variant, having previously undergone multiple therapies, was treated with olaparib and cisplatin, resulting in progression-free survival for 12 months.^111^ No clinical trials using PARP inhibitors have focused on HCC.

## Rare Germline Variants That Are Protective of Hepatocellular Carcinoma

The loss of function in the *HSD17B13* SNP rs72613567 has been associated with a decreased risk of liver diseases across 3 European studies. The initial study^112^ conducted WES in both discovery and replication phases, encompassing 46,544 patients with alcoholic and nonalcoholic liver disease, cirrhosis, and HCC, each matched with controls. A replication phase included 12,527 patients drawn from populations in the United States. In the discovery phase, the SNP was associated with diminished risk of alcoholic liver disease and MASLD, and cirrhosis from these conditions. The protection by this SNP extended to HCC risk (OR, 0.67; 95% CI, 0.45–1; *P* = .047). In the replication phase, decreased risk was confirmed for liver diseases in general. Two other multicenter studies confirmed that the SNP decreases the risk of HCC in patients with alcoholic liver disease^113^^,^^114^ (OR, 0.59; 95% CI, 0.44–0.79 and OR, 0.77; 95% CI, 0.68–0.89, respectively). In addition, in patients who harbor the higher risk rs738409 allele in *PNPLA3*, the variant in *HSD17B13* attenuated the risk of HCC (OR, 0.75; 95% CI, 0.64–0.87).^114^

HSD17B13 is an enzyme implicated in fatty acid metabolism and the biological activity of sex hormones.^115^ In mouse models, the enzyme is up-regulated in fatty liver and localizes to liver lipid droplets.^116^ The precise mechanism governing the enzyme’s loss of function and its correlation with a reduced risk of developing HCC needs further investigation.

Loss-of-function variants in *CIDEB* were associated with a reduced risk of liver disease of any cause (OR, 0.67; 95% CI, 0.57–0.79) and lower risk of HCC (OR, 0.51; 95% CI, 0.26–1) in an exome-wide association analysis that compared participants with various types of liver diseases with controls without liver disease, analyzing multiple biobanks in Europe, United States, and UK.^117^ *CIDEB*, or cell death-inducing DFFA-like effector B, is a member of the CIDE family, which is associated with lipid clustering and fusion.^118^^,^^119^ *CIDEB* is expressed in the liver and exhibits a correlation with very-low-density lipoprotein maturation. In mice models, in vitro overexpression of *CIDEB* demonstrated fat accumulation in larger lipid droplets, whereas knockout mice exhibited hepatocytes with smaller droplets.^117^ Further investigation is warranted to elucidate the mechanisms of CIDEB in lipid droplet formation and to understand how its loss of function may decrease liver disease risk and, consequently, HCC risk.

## Genetic Associations for Cholangiocarcinoma

CCA is classified on the basis of its anatomic location as intrahepatic, perihilar, or distal to the biliary tree. There have not been any large-scale GWAS of CCA that have been published to date, but there are ongoing consortia that are actively pursuing this.^120^^,^^121^

The yield of genetic testing for rare variants in cancer-associated genes has been similar across different populations of patients with CCA (Figure 1*B*). Several studies were conducted in Chinese populations. A study that included 122 patients with IHC found P/LP germline variants in 6.56%, including in *BRCA1*, *BRCA2*, *FANCA*, and *MLH1* genes.^102^ Another study that involved a total of 382 patients with BTC found P/LP germline variants in moderate and high penetrance genes *ATM, BLM, BRCA2, ERCC2,* and *RAD54L*.^122^ A study of 188 patients with CCA found 5.8% had germline variants in *ATM, BLM, BRCA2, ERCC5, MSH2, PALB2, RAD50,* and *XPC*.^123^

Two studies conducted in the United States have investigated rare variants in cancer-associated genes among patients with BTC. The first study included a panel of 88 genes in 131 patients with BTC from a cancer center in New York.^124^ Sixteen percent of the patients carried germline P/LP variants, including 7 patients with high penetrance genes in *BAP1*, *BRCA1, BRCA2*, *PALB2,* and *PMS2* in IHC patients, whereas *BRCA1* was the only high penetrance gene found in EHC patients. The second study was a multicenter study in the United States that examined variants in 83 genes in 205 patients with HBC.^101^ There were 14.7% of patients with IHC who were found with variants, including in *BRCA1*, *BRCA2*, *MLH1*, and *TP53,* and 5.8% with EHC variants, including in *BRCA2* and *TP53*.

A study sequenced a panel of 49 genes in a cohort of 146 Japanese patients with BTC.^125^ They found that 11% of those 146 patients had P/LP germline variants, including 6 patients with IHC carrying variants in *BRCA1*, *BRCA2*, *MLH1*, and *MSH2*; 3 patients with perihilar CCA carrying variants in *BRCA2*, *MLH1*, and *POLE*; and 6 patients with EHC carrying variants in *ATM, BRCA2*, *MLH1, POLD1*, and *RAD51D*. In another Japanese population study, whole genome sequencing was performed on 1292 patients with BTC, including IHC, EHC, gallbladder cancer (GBC), and ampulla of Vater carcinoma. Germline variants in high penetrance genes *ATM, BRCA1/2, BRIP1, MLH1, MSH2* and *MSH6* were found in patients with IHC and EHC.^126^

Pathogenic germline variants in *BRCA1* and *BRCA2* are prevalent in patients with CCA, but the pathogenesis of *BRCA*1/2 variants in driving this cancer type and the role of targeted therapy such as PARP inhibitors remain poorly studied in CCA compared with other cancer types such as ovarian and breast cancer.^127^ A study involving Chinese patients with IHC treated 7 patients with *BRCA2* mutations detected in tumor tissues, 3 of which were in the germline, with the PARP inhibitor olaparib.^102^ The study found that the 3 patients with germline mutations in *BRCA2* achieved a partial response, whereas the remaining 4 with somatic mutations in *BRCA2* achieved a stable response or experienced progressive disease. In larger study conducted in Spain and Italy, 72 patients with *BRCA1/2* mutations in IHC tumor tissue and 78 without were treated with platinum-based chemotherapy. There was improved progression-free survival in patients with *BRCA1/2* variants compared with those without.^128^ Ongoing clinical trials are exploring the treatment of BTCs with BRCA mutations (NCT03337087,^129^ NCT03639935,^130^ NCT04042831,^131^ and NCT04306367^132^). If results demonstrate efficacy of PARP inhibitors in BTCs, it will likely lead to broader adoption of germline and tumor genetic testing for individualized therapy for CCA.

## Genetic Associations for Gallbladder Cancer

Gallbladder cancer incidence has varying geographical distributions, with the highest average incidence rates observed in Bolivia (21 per 100,000), Chile (11.7 per 100,000), Bangladesh (7.3 per 100,000), Nepal (6 per 100,000), and Peru (6 per 100,000). Unlike HCC and CCA, women have approximately twice the likelihood of developing GBC compared with men.^28^^,^^133^

GWAS have investigated SNPs associated with GBC risk (Table 2). A study conducted in Japan included 41 GBC cases and 866 disease-free controls in the discovery phase and 30 GBC cases and 898 disease-free controls in the validation phase.^134^ The study identified SNP rs7504990 within the *DCC* gene was enriched in GBC cases compared with controls (OR, 6.95; 95% CI, 3.43–14.08). *DCC* encodes the netrin1-receptor, a protein that plays a role in a family of cell adhesion molecules.^135^ The *DCC* gene is well-documented as a tumor suppressor and has been documented to undergo loss of heterozygosity in other gastrointestinal cancer types such as colon,^136^ esophagus,^137^ stomach,^138^ and pancreas.^139^ Loss of *DCC* has been hypothesized to reduce cell adhesion and increase risk of cancer progression and metastasis.^139^ Additional multiethnic studies are required to confidently classify the association for GBC and to guide surveillance recommendations for people at risk.

**Table 2:** List of Genome-wide Association Studies of GBC

Gene	SNP	Risk allele	OR (95% CI)	*P* value	Type of cancer cases	Type of controls	No. of subjects				Ethnic group/country	References
							Discovery phase		Replication phase			
							Cases	Controls	Cases	Controls		
*ABCB1*	rs17209837	A	1.61 (1.38–1.89)	2.26 × 10^−9^	GBC	Healthy	1042	1709	428	420	Indian	134
*ABCB4*	rs1558375	A	1.47 (1.30–1.66)	2.31 × 10^−10^	GBC	Healthy	1042	1709	428	420	Indian	134
	rs4148808	A	1.57 (1.35–1.82)	2.71 × 10^−^⁹								
*DCC*	rs7504990	A	6.95 (3.43–14.08)	7.46 x 10^−8^^a^	GBC	Healthy	41	866	30	898	Japanese	128

GBC, gallbladder cancer.

a Study that has a *P* value greater than 5 × 10^−8^.

A second GWAS study conducted in India included a discovery phase with 1042 GBC cases and 1709 healthy controls and a validation phase with 428 GBC cases and 420 healthy controls.^140^ The study identified several significant SNPs located within the chromosomal region 7q21.12, specifically SNP rs17209837 within the *ABCB1* (OR, 1.61; 95% CI, 1.38–1.89) and SNPs rs1558375 (OR, 1.47; 95% CI, 1.30–1.66) and rs4148808 (OR, 1.57; 95% CI, 1.35–1.82) within the *ABCB4* gene loci. Both the *ABCB1* and *ABCB4* genes belong to the ATP-binding cassette transporter family. The *ABCB4* gene encodes a transporter of phospholipids across hepatocyte membranes, releasing these phospholipids into bile acids.^141^ *ABCB1* encodes P-glycoprotein, which functions to pump xenobiotics, including toxins and drugs, out of cells.^142^ In the context of GBC, decreased *ABCB4* expression may increase reactive oxygen species and lipid peroxidation within gallbladder epithelial cells, resulting in inflammation and DNA damage.^141^ Meanwhile, *ABCB1* overexpression is associated with cancer drug resistance.^142^ The combination of these SNPs may contribute to gallbladder carcinogenesis, but study in additional populations is needed for generalizability.

A third GWAS was conducted in Chile mainly focused on gallbladder disease while conducting a detailed subanalysis on individuals who developed GBC.^143^ Within the subanalysis, the study used 397 GBC cases and 667 controls, all of whom were female. The study unveiled nominal associations between gallstone disease and GBC and SNPs at 2 specific gene loci, *ABCG8* and *TRAF3*. However, the results did not reach genome-wide significance.

Several studies in GBC have examined the prevalence of rare germline variants in cancer-associated genes such as DDR genes (Figure 1*C*). At a cancer center in New York, researchers found 5 of the 22 patients (22.7%) with GBC had a pathogenic germline variant, 3 with *BRCA2*, and 1 each with an *APC* and *NBN* variants.^124^ In a study conducted in China 13 of the 164 GBC cases had heterozygous P/LP germline variants (7.9%), including in *ATM*, *BRCA1*, *EPCAM*, *FANCA*, *FANCC*, *MLH, MUTYH, RAD50*, *RAD51D,* and *SPINK1*.^144^ A separate study from China included 117 patients with GBC and found germline variants in 11 patients (9.4%), including in *ATR*, *BLM, BRCA2*, *BRIP1, ERCC1, FANCC, PALB2*, *RAD54L*, *RECQL4*, and *XPA*.^122^ Another study conducted in Japan performed targeted sequencing in 27 cancer-predisposing genes in a cohort of 219 patients with GBC and found that ∼5.5% of 219 patients with GBC had a P/LP germline variant, including in *BRCA1*, *BRCA2*, *BRIP1*, *MLH1*, *MSH6*, *NBN*, *RAD51D*, and *TP53*.^126^

Overall, *BRCA1* and *BRCA2* mutations have been reported in multiple studies of GBC. Supporting a role for *BRCA1/2* in GBC, the Breast Cancer linkage consortium studied coincident cancers among 681 individuals with breast or ovarian cancer and 3047 carriers of *BRCA1* or *BRCA2* variants. They found a relative risk for gallbladder and bile duct cancer of 4.97 (95% CI, 1.50–16.52).^145^

## Conclusions and Future Directions

Patients with hepatobiliary cancers bear a disproportionate burden of cancer-related morbidity and mortality.^1^ There has been significant progress in discovering SNPs and P/LP germline variants associated with increased risk of developing HBCs. However, these studies have limitations, and broad implementation of genetic testing in HBC requires further investigation.

Several GWAS have found SNPs at *TM6SF2*^70^^,^^69^ and *PNPLA3*^70^^,^^69^^,^^72^^,^^71^ are consistently associated with hepatocarcinogenesis in patients with MASLD or chronic alcohol consumption. Other SNPs lack independent confirmation, which may stem from population or risk factor differences, insufficient statistical power, or minor impact of individual SNPs on HCC. Recently, risk stratification studies have implemented PRS that incorporate metabolic-associated SNPs to assess their cumulative genetic influence on HCC susceptibility.^146^, ^147^, ^148^, ^149^ These studies revealed that PRS independently does not outperform traditional clinical risk score. However, in combination with the clinical score model, it marginally performs better than a clinical risk score in isolation.^146^, ^147^, ^148^, ^149^ This may, in part, be related to overlap in what the PRS and clinical score are measuring, risk of worsening of liver disease. Perhaps inclusion of variants more directly linked to cancer initiation such as SNPs at the *WNT* or *TERT* loci and rare variants in cancer-associated genes will improve genetic risk assessment. Larger, more diverse studies are needed to find new variants and improve PRS performance.^150^ HCC GWAS have yet to adequately explore genetic associations in underrepresented groups such as Africans and Latin Americans.^151^, ^152^, ^153^

GWAS studies for CCA are in process and promise to include multiethnic international populations. It will be interesting to know whether SNPs associated with HCC or GBC risk are also associated with CCA, given overlaps in the tissue of origin and in some environmental risk factors.

In GBC, several SNPs have been reported to be associated with risk in relatively small cohorts. There is a need for confirmation across populations and for larger, multiethnic GWAS. This is challenging because of the rarity of GBC.

Many of the studies on rare variants in cancer-associated genes reviewed here have limitations. Most did not exclusively study HCC, CCA, or GBC and instead combined these heterogenous cancers together. In addition, they generally do not compare rates of P/LP variants with non-cancer controls selected from the same population. This is important because it is currently unclear whether specific hereditary cancer syndromes are more prevalent in HBC than in cancer-free populations. Apart from a single study on metabolic-associated genes, all the rare variant analysis done on HBC patients has focused on cancer-related genes that were established in studies focused on other cancer types. Future studies should use exome-wide analysis to examine whether other genes may be linked to risk of HBC. In addition, rare variant analysis studies should use established criteria for variant calling and should report the variant sequences in publications or in publicly accessible databases.^39^^,^^154^ The ultimate goal will be to determine, on the basis of genetic factors plus environmental exposures, whether certain subgroups of patients are more susceptible to developing HBC. Finally, cases and controls should include underrepresented populations to determine which variants are truly pathogenic, because variant allele frequency is often unknown in understudied populations.

Organ transplantation can be the only chance for a durable cure for patients with HCC and CCA who meet certain criteria.^155^^,^^156^ It is important to note that genetic testing results should not negatively impact the access to transplantation for patients. The primary goal of genetic testing in the context of HBC is to identify hereditary cancer syndromes or variants that can be targeted with new therapies and to test other family members at risk.^39^ Patients with P/LP variants in *BRCA1*, *BRCA2*, or Lynch syndrome carriers face lifetime cancer risks of 20%–80%, including breast, ovarian, colon, and endometrial cancer.^38^^,^^40^ Patients with HBC discovered to have an inherited cancer predisposition variant, especially those in remission, should meet with an expert in cancer genetics to discuss risk mitigation strategies such as screening with mammography and colonoscopy and risk-reduction surgery. Detecting these genetic variants could potentially lead to a shift in clinical practices, enabling precision medicine treatments that extend the survival of patients with HBC.
